# Management and treatment outcomes of rheumatoid arthritis in the era of biologic and targeted synthetic therapies: evaluation of 10-year data from the KURAMA cohort

**DOI:** 10.1186/s13075-023-03251-z

**Published:** 2024-01-09

**Authors:** Takayuki Fujii, Koichi Murata, Hideo Onizawa, Akira Onishi, Masao Tanaka, Kosaku Murakami, Kohei Nishitani, Moritoshi Furu, Ryu Watanabe, Motomu Hashimoto, Hiromu Ito, Takao Fujii, Tsuneyo Mimori, Akio Morinobu, Shuichi Matsuda

**Affiliations:** 1 Department of Advanced Medicine for Rheumatic Diseases, Graduate School of Medicine, Kyoto University, 54 Shogoin Kawaharacho, Sakyo, Kyoto, 6068507 Japan; 2 Department of Orthopaedic Surgery, Graduate School of Medicine, Kyoto University, 54 Shogoin Kawaharacho, Sakyo, Kyoto, 6068507 Japan; 3 Center for Cancer Immunotherapy and Immunobiology, Graduate School of Medicine, Kyoto University, 54 Shogoin Kawaharacho, Sakyo, Kyoto, 6068507 Japan; 4 Furu Clinic, 1098 Terasho, Konancho, Koka, Shiga 5203301 Japan; 5 Department of Clinical Immunology, Osaka Metropolitan University Graduate School of Medicine, 1-5-7 Asahicho, Abeno, Osaka, 5450051 Japan; 6 Department of Orthopaedic Surgery, Kurashiki Central Hospital, 1-1-1 Miwa, Kuchiki, Okayama, 7100052 Japan; 7 Department of Rheumatology and Clinical Immunology, Wakayama Medical University, 811-1 Kimiidera, Wakayama, 6410012 Japan; 8 Takeda Clinic for Rheumatic Diseases, 606-3-2, Higashi-Shiokojicho, Sanoh Kyotoekimae Building 1F, Kyoto, 6008216 Japan; 9 Department of Rheumatology and Clinical Immunology, Graduate School of Medicine, Kyoto University, 54 Shogoin Kawaharacho, Sakyo, Kyoto, 6068507 Japan

**Keywords:** Rheumatoid arthritis, Cohort study, Biologics, JAK inhibitors, Treatment outcomes

## Abstract

**Background:**

Advances in rheumatoid arthritis (RA) treatment, highlighted by biological disease-modifying antirheumatic drugs (bDMARDs) and targeted synthetic DMARDs (tsDMARDs), have altered the paradigm of RA treatment in the last decade. Therefore, real-world clinical evidence is needed to understand how treatment strategies and outcomes have changed.

**Methods:**

Using an observational cohort of RA from 2012 to 2021, we collected cross-sectional data of RA patients annually to analyze a trend in RA management. For patients who initiated b/tsDMRDs, we evaluated treatment outcomes between b/tsDMARDs. Mixed-effect models were applied to examine the statistical implications of changes over time in treatment outcomes with a background adjustment.

**Results:**

We analyzed annual cross-sectional data from 5070 patients and longitudinal data from 1816 patients in whom b/tsDMARDs were initiated between 2012 and 2021. b/tsDMARD use increased, whereas glucocorticoid use decreased from 2012 to 2021. Disease activity and functional disability measures improved over time. The percentage of tsDMARD prescriptions considerably increased. All b/tsDMARDs showed clinical improvements in disease activity and functional disability. Statistically, TNFi showed better short-term improvements in b/tsDMARD-naïve patients, while IL6Ri demonstrated significant long-term benefits. IL6Ri had better retention rates in switched patients. After adjustment for patient characteristics, the annual change of RA disease activity and functional disability fared significantly better from 2012 to 2021.

**Conclusions:**

With the development of new RA therapeutics, overall treatment outcomes advanced in the past decade.

**Supplementary Information:**

The online version contains supplementary material available at 10.1186/s13075-023-03251-z.

## Background

Treatment options for rheumatoid arthritis (RA) have progressed over the past two decades. Methotrexate (MTX), biologic disease-modifying antirheumatic drugs (bDMARDs), and targeted synthetic antirheumatic drugs (tsDMARDs) such as Janus kinase inhibitors (JAKis) have significantly improved disease activity, functional disability, and joint prognosis of RA patients [1, 2]. This clinical evidence is primarily based on well-designed randomized controlled trials; however, they cannot assess long-term efficacy and safety outside of the study period or advocate evidence in the groups of patients who met exclusion criteria due to age or comorbidities [3, 4]. Real-world data obtained from observational cohort studies could not only answer these questions but also describe how patient backgrounds and therapeutic methods affected treatment outcomes in the observational period [5].

The Kyoto University Rheumatoid Arthritis Management Alliance (KURAMA) cohort is an open-label, single-center, observational cohort study of RA and rheumatic diseases [6–8]. It was established in 2011, and a total of 4418 patients were registered until 2022. We recorded longitudinal treatment data at every clinical visit, including disease activity, functional disability, and adverse events. We also have conducted the “annual RA survey” that features radiographic examinations on joint destruction, testing for osteoporosis, and patient surveys regarding frailty, sarcopenia, mental status, and more every year since 2012 [9–12]. The KURAMA cohort also has a biobank of plasma, synovium, and other RA-related specimens [13–15].

Between 2012 and 2021, the Japanese Ministry of Health, Labour and Welfare approved two bDMARDs (certolizumab pegol, 2013; sarilumab, 2018) and five JAKis (tofacitinib, 2013; baricitinib, 2017; upadacitinib; peficitinib; and filgotinib, 2021) [16]. This study aimed to investigate how treatment strategies and outcomes evolved alongside advances in therapeutic strategies by reviewing the 10-year experience of the KURAMA cohort from 2012 to 2021.

## Materials and methods

### Patients

To observe the annual trends in RA management, we gathered clinical data from RA patients, including disease activity, functional disability, the usage of MTX, glucocorticoids (GCs), and b/tsDMARDs, along with demographic and anthropometric details, from the annual RA surveys conducted from 2012 to 2021. For patients treated with b/tsDMARDs, we recorded disease activity; functional disability before and 1, 2, 3, 6, and 12 months after initiation; and the date of initiation and discontinuation. Disease activity was assessed using the Disease Activity Score (DAS) 28-CRP and Clinical Disease Activity Index (CDAI), and functional disability was evaluated by the Health Assessment Questionnaire Disability Index (HAQ) [17, 18]. All patients met the 1987 American College of Rheumatology (ACR) classification criteria or the 2010 ACR/European League Against Rheumatism classification criteria for RA diagnosis. The KURAMA cohort study was approved by the Kyoto University Graduate School of Medicine Medical Ethics Committee (R0357), and informed consent was obtained from all patients.

### Data analysis

Since the annual cross-sectional dataset had relatively low missing value rates, with the highest rate at 8.4% for DAS28-CRP, data with missing values were excluded from subsequent statistical analyses.

We employed propensity score matching to address potential confounding factors that could impact the effectiveness of b/tsDMARDs. The reference group consisted of patients treated with CTLA4-Ig (abatacept). The following variables were used to account for potential confounding factors: age, sex, CDAI, DAS28-CRP, HAQ, disease stage, disease class, methotrexate (MTX) and glucocorticoid (GC) dosages, rheumatoid factor (RF), and anti-cyclic citrullinated peptide antibody (ACPA) titers at baseline. One-to-one matching without replacement was performed using the nearest neighbor match on the logit of the propensity score with the caliper width set to the standard deviation of the logit of the propensity score. Student’s *t*-test and the chi-square test were used to analyze continuous variables and categorical variables, respectively.

For the time-series analysis, we fitted mixed-effects models to evaluate the trends in disease activity and functional disability measures [19]. All models were fitted with patient-specific random intercepts to account for inter-patient variations. Box-Cox transformation and square root transformation were applied to CDAI and HAQ, respectively, to fix skewness and gain a better fitting [20, 21]. Age, sex, BMI, RF, ACPA, Steinbrocker’s stage, class, dose of MTX, and dose of GCs were used as covariates.

Statistical analyses were performed using scipy v1.7.1, statsmodels v0.12.2, and scikit-learn v0.24.2. Propensity score matching was performed using psmpy v0.3.13. Kaplan‒Meier survival estimation was performed using lifelines v0.27.3 running on Python v3.9.7. We used ggalluvial v0.12.5 for the Sankey diagram, car v3.1–2 for Box-Cox transformation, and glmmTMB v1.1.7 for a mixed-effect model, running on R v4.2.0. Graphs were drawn using matplotlib v3.4.3, seaborn v0.11.2, and ggplot2 v3.4.2.

## Results

### Changes in RA patient background and RA treatment trends from 2012 to 2021

**Fig. 1:**
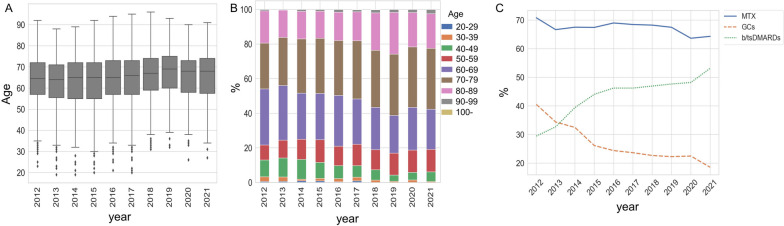
Change in patients’ demographics and treatment from 2012 to 2021. **A** Box plots showing the change in the mean patient age. **B** Change in the proportion of age groups. **C** Percentage of patients treated with MTX, GCs, and b/tsDMARDs

From 2012 to 2021, a total of 1156 patients and a cumulative total of 5070 patients participated in annual RA surveys. The mean age of RA patients increased from 62.9 in 2012 to 65.9 in 2021, and the percentage of patients younger than 60 years old was less than 20% in 2021 (Fig. 1A, B, and Supplementary Table S1). This trend may reflect the aging society of Japan. Regarding treatment, the percentage of patients treated with MTX was over 60% in all periods and slightly decreased between 2012 and 2021 from 70.8 to 64.3% (Fig. 1C). GC usage decreased (40.5–18.6%), whereas b/tsDMARD usage (29.5–53.2%) increased, indicating that more intensive treatment has been administered to RA patients in the past decade (Fig. 1C). The patients’ demographics and treatment methods during the observation period are summarized in Supplementary Table S1.

### The transition of disease activity and functional disability in 10 years

**Fig. 2:**
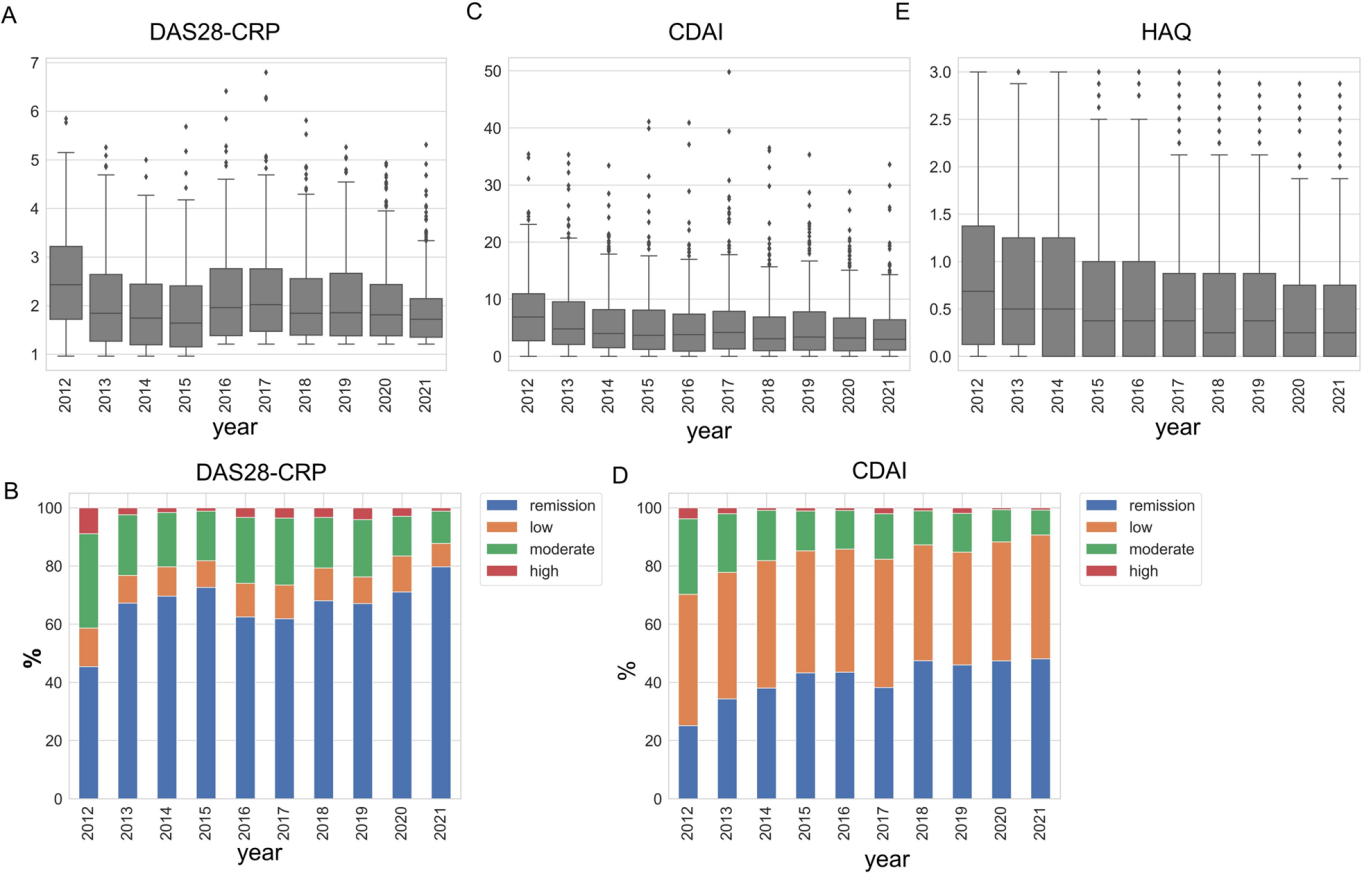
Evolution of disease activity and functional disability over 10 years. **A** Change in DAS28-CRP. **B** Proportion of disease activity categories defined by DAS28-CRP. **C** Change in CDAI. **D** Proportion of disease activity categories defined by CDAI. **E** Change in HAQ

We next investigated how disease activity and functional disability changed over 10 years. The average DAS28-CRP sharply decreased from 2012 to 2015, during which the number of patients treated with b/tsDMARDs increased (Fig. 1C) and then remained stable thereafter (Fig. 2A and Supplementary Table S2). The percentage of patients who achieved DAS28-CRP remission gradually increased and reached 79.7% in 2021 (Fig. 2B). Similarly, CDAI decreased, and the rate of CDAI remission also increased from 25.1 to 48.1% in 10 years (Fig. 2C, D, and Supplementary Table S2). Moreover, the median HAQ scores decreased over time from 0.69 to 0.25 (Fig. 2E and Supplementary Table S2).

### Treatment outcomes of b/tsDMARDs in the KURAMA cohort

Because an increasing number of patients in the KURAMA cohort were treated with b/tsDMARDs and achieved good clinical outcomes from 2012 to 2021, in which several new b/tsDMARDs were approved, we next analyzed the real-world treatment outcomes of b/tsDMARDs using longitudinal observational data on the treatment responses to b/tsDMARDs. We found 1816 new prescriptions (820 for b/tsDMARD-naïve patients and 996 for b/tsDMARD-switching patients) between 2012 and 2021. Baseline demographic data are shown in Supplementary Table S3; the patients in this dataset partially overlap with those in Supplementary Tables S1 and S2. Supplementary Fig. S1 shows the trends in the proportion of initiated b/tsDMARDs categorized by mode of action (MOA) from 2012 to 2021. The proportion of IL-6 receptor inhibitors (IL6Ri; tocilizumab and sarilumab) was almost consistent over 10 years, accounting for approximately 10% of naïve cases and 20% of switch cases. The initiation of TNF inhibitors (TNFi; infliximab, etanercept, adalimumab, golimumab, and certolizumab pegol, including their biosimilars) and CTLA4-Ig decreased in both types of cases from 2012 to 2021. The prescription of JAKis increased from 2014 in switch cases first, and JAKis were more recently initiated in over 20% of naïve patients (Supplementary Fig. S1). Concomitant usage of MTX in b/tsDMARD-naïve patients and switched patients was 74.5% and 63.3% in TNFi, 40.7% and 56.6% in IL6Ri, 47.3% and 48.5% in CTLA4-Ig, and 62.0% and 47.0% in JAKi, respectively (Supplementary Table S3).

We next compared the effectiveness of b/tsDMARDs, assessing the transition of DAS28-CRP, CDAI, and HAQ as depicted in Supplementary Fig. S2. Each MOA achieved low disease activity or remission according to the averaged DAS28-CRP and CDAI at 6 months (Supplementary Fig. S2). Similarly, in switched cases, all MOAs achieved CDAI levels below the threshold for low disease activity at 12 months (Supplementary Fig. S2). To address potential confounding factors, we applied propensity score matching, with the CTLA-4 group as the reference group, to evaluate the drug effectiveness in comparison to CTLA-4. Notably, we excluded JAKis from b/tsDMARDs-naïve cases, as the number of patients treated with JAKis as a first-line therapy was fewer than other MOAs (Supplementary Table S3). In b/tsDMARDs-naïve patients, TNFi showed a statistically better improvement in CDAI and DAS28-CRP at 1 and 2 months, and IL6Ri significantly ameliorated CDAI at 2, 6, and 12 months, and DAS28-CRP at 1, 2, 6, and 12 months compared to CTLA4-Ig (Table 1). All MOAs exhibited gradual improvements in HAQ, with no statistical differences (Table 1). In switched cases, all measures of disease activity and functional disability were comparable across all comparisons (Table 1).

**Table 1:** *P*-values computed to compare the effectiveness of b/tsDMARDs in the propensity score-matched groups

Month	DAS28-CRP						CDAI						HAQ					
	Baseline	1	2	3	6	12	Baseline	1	2	3	6	12	Baseline	1	2	3	6	12
Naïve																		
TNFi (*n* = 84) vs CTLA4-Ig (*n* = 84)	0.8895	0.0009	0.0379	0.2770	0.0644	0.1696	0.9738	0.0012	0.0241	0.2069	0.0522	0.0734	0.9555	0.2426	0.3540	0.6164	0.0995	0.2158
IL6Ri (*n* = 42) vs CTLA4-Ig (*n* = 42)	0.3924	0.0052	0.0006	0.0581	0.0017	0.0002	0.4478	0.1712	0.0377	0.4847	0.0339	0.0021	0.5205	0.1566	0.1634	0.9074	0.0684	0.0855
Switch																		
TNFi (*n* = 66) vs CTLA4-Ig (*n* = 66)	0.7118	0.3973	0.7034	0.8598	0.7530	0.5893	0.9324	0.8345	0.5103	0.4629	0.6256	0.9206	0.6335	0.3683	0.1135	0.3937	0.3156	0.2418
IL6Ri (*n* = 65) vs CTLA4-Ig (*n* = 65)	0.6384	0.1762	0.9128	0.4938	0.9962	0.2088	0.8586	0.5670	0.1929	0.2623	0.2064	0.9789	0.5369	0.2064	0.1017	0.3292	0.0777	0.1196
JAKi (*n* = 49) vs CTLA4-Ig (*n* = 49)	0.8015	0.3209	0.4526	0.7115	0.6972	0.1140	0.9185	0.9117	0.7453	0.3853	0.7996	0.6086	0.3997	0.5394	0.4940	0.9882	0.2413	0.2345

*TNFi* TNF inhibitors, *IL6Ri* IL-6 receptor inhibitors, *CTLA4-Ig* abatacept, *JAKi* Janus kinase inhibitors, *DAS28-CRP* Disease Activity Score 28-C-reactive protein, *CDAI* Clinical Disease Activity Index, *HAQ* Health Assessment Questionnaire Disability Index

**Fig. 3:**
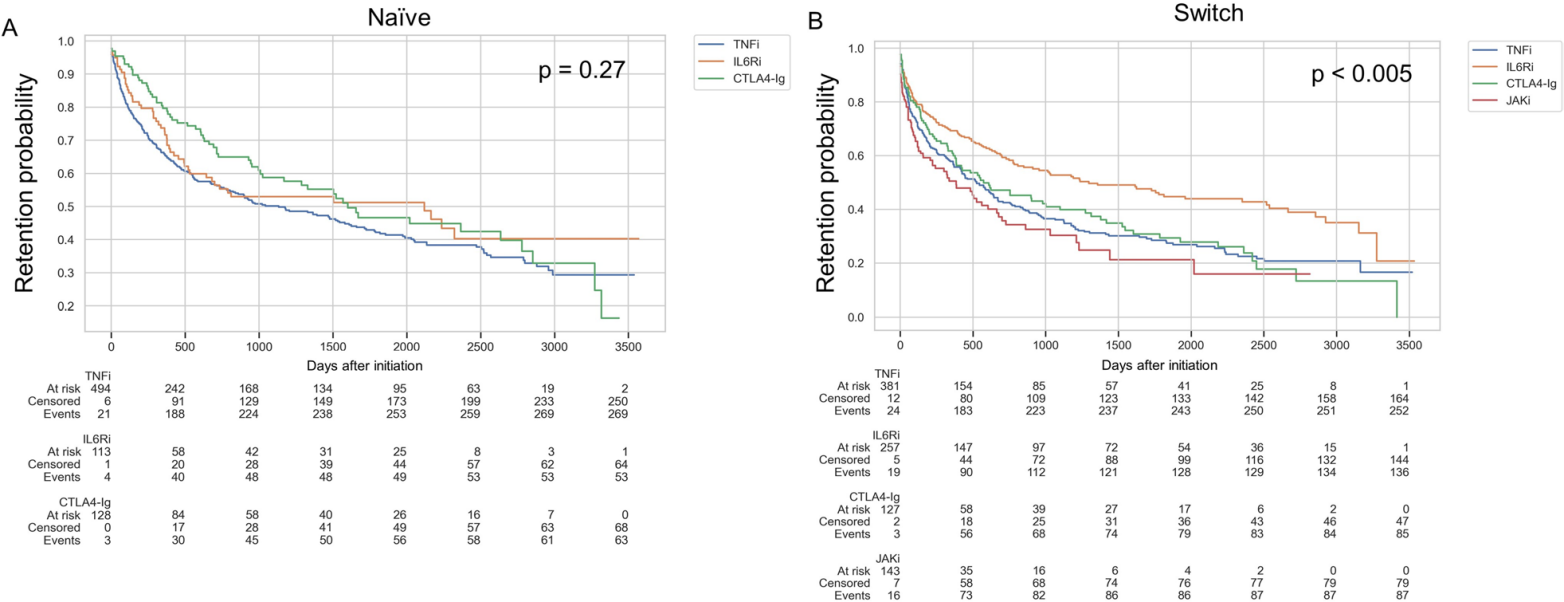
Drug retention rate of b/tsDMARDs. **A**, **B** Kaplan–Meier survival curves showing drug retention comparing different MOAs. The *X*-axis shows the number of days after b/tsDMARDs initiation, and the *Y*-axis shows the retention probability. Statistics used: log-rank test. **A** Drug retention in b/tsDMARD-naïve patients. **B** Drug retention in switched patients

The retention of b/tsDMARDs is another indicator of successful treatment [22–25]. For Kaplan‒Meier analysis, we defined an event as when the prescription of b/tsDAMRDs ceased due to inefficacy and adverse events, and discontinuation due to remission, socioeconomic issues, or the patient’s decision was defined as a censored case. There was no significant difference in the retention rate in b/tsDMARD-naïve patients (Fig. 3A). Moreover, IL6Ri had a significantly better retention rate (*p* < 0.005) in switched patients (Fig. 3B), which agrees with a previous report [23]. The MOA and continuation/discontinuation status of the first b/tsDMARDs in naïve patients and the choice of MOA when switching are summarized in the Sankey diagram (Supplementary Fig. S3). Different MOAs tended to be chosen when switching from IL6Ri; however, the same MOA was selected in approximately half of the cases when switching from JAKi and TNFi (Supplementary Fig. S3). These results show treatment outcomes of b/tsDMARDs between different MOAs in a real-world setting.

### Statistical implications of the annual trends in disease activity and functional disability

**Fig. 4:**
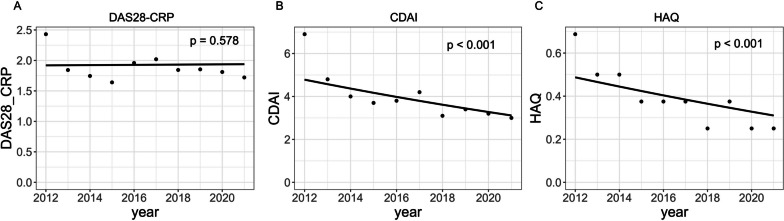
Mixed-effect model fitted to the annual change in disease activity and functional disability. **A**–**C** The dots denote the observed median values, and the lines represent the regression line fitted using mixed-effect models. **A** DAS28-CRP. **B** CDAI. **C** HAQ

To know the statistical implications of observed trends in DAS28-CRP, CDAI, and HAQ from 2012 to 2021 (Fig. 2A–D), we used a mixed-effect model for adjusting patient-specific characteristics [19]. The annual changes in CDAI and HAQ were statistically significant, but not DAS28-CRP (Fig. 4A–C and Table 2). Patients’ demographic data such as age, stage, and class significantly affected HAQ, and b/tsDMARDs, MTX, and GC use were significantly associated with DAS28-CRP and CDAI. b/tsDMARDs use was also associated with HAQ (Table 2).

**Table 2:** Results of the mixed-effect model

	DAS28-CRP				CDAI				HAQ			
	Estimate	Std. error	*z* value	Pr( >|*z*|)	Estimate	Std. error	*z* value	Pr( >|*z*|)	Estimate	Std. error	*z* value	Pr( >|*z*|)
(Intercept)	− 1.623	2.914	− 0.557	0.57761	142.9	15.8	9.048	< 0.0001	31.03	3.191	9.725	< 0.0001
year	0.000827	0.00145	0.57	0.5684	− 0.0715	0.00787	− 9.083	< 0.0001	− 0.0157	0.0016	− 9.831	< 0.0001
Age	0.00126	0.00052	2.416	0.01571	0.00616	0.00319	1.932	0.05335	0.01008	0.00087	11.538	< 0.0001
Sex	0.01499	0.01745	0.859	0.39008	0.2061	0.1073	1.921	0.05473	0.1302	0.02968	4.388	< 0.0001
Stage	0.05452	0.00498	10.943	< 0.0001	0.3038	0.0289	10.512	< 0.0001	0.06813	0.00638	10.674	< 0.0001
Class	0.09475	0.00698	13.57	< 0.0001	0.6173	0.03747	16.476	< 0.0001	0.1606	0.00738	21.768	< 0.0001
b/tsDMARDs use	− 0.0546	0.01083	− 5.041	< 0.0001	− 0.1233	0.06147	− 2.006	0.04485	0.05008	0.01282	3.908	< 0.0001
dose_MTX	0.00366	0.00121	3.036	0.0024	0.02375	0.00676	3.511	0.00045	3.6E − 05	0.00138	0.026	0.97893
dose_GC	0.00346	0.00102	3.389	0.0007	0.01246	0.0054	2.309	0.02096	0.00156	0.00099	1.579	0.11423
BMI	0.00369	0.00165	2.235	0.02544	0.00605	0.00969	0.625	0.53224	− 0.0008	0.00223	− 0.363	0.71695
RF	9.5E − 05	1.6E − 05	5.763	< 0.0001	0.00047	9E − 05	5.252	< 0.0001	5.9E − 05	1.8E − 05	3.385	0.00071
ACPA	3.3E − 05	1.2E − 05	2.782	0.0054	0.0001	6.6E − 05	1.569	0.11676	1E − 05	1.3E − 05	0.777	0.43714

*DAS28-CRP* Disease Activity Score 28-C-reactive protein, *CDAI* Clinical Disease Activity Index, *HAQ* Health Assessment Questionnaire Disability Index, *RF* rheumatoid factor, *ACPA* anti-citrullinated protein antibody, *MTX* methotrexate, *GC* glucocorticoid, *b/tsDMARDs* biologic or targeted synthetic disease-modifying antirheumatic drugs, *BMI* body mass index

## Discussions

RA treatment strategies have evolved over the past 20 years. Two bDMARDs and five tsDMARDs were launched in the last decade, and several drugs with novel MOAs are in clinical trials. Therefore, tracking the RA management of individual patients and analyzing real-world data is crucial for addressing whether the management of RA has improved in the real-world setting. In this study, we analyzed the trends and outcomes of RA treatments in the KURAMA cohort between 2012 and 2021; we showed that (1) the percentage of patients who were treated with GCs and b/tsDMARDs changed significantly, (2) treatment responses and drug retentions differed between different MOAs, and (3) metrics of disease activity and functional disability improved over time. Here, we demonstrated the disparity in b/tsDMARD effectiveness and yearly improvements in the overall disease activity and functional disability using propensity score matching and mixed-effects modeling to account for patient-specific factors.

We found that GC use consistently decreased while b/tsDMARD use increased over a decade, which is consistent with other studies that observed real-world trends of RA medications [5, 26]. However, some reported that the proportion of patients treated with b/tsDMARDs or GCs was stable over the observation period [16, 27]. Regarding treatment outcomes, studies have shown a stable decrease in disease activity and functional disability indices from the 1990s or early 2000s to the early 2010s [5, 27–32]. In contrast, some studies showed that the average DAS28 or DAS28 remission rate remained the same after the early 2010s, which is consistent with our data [5, 28]. We found that CDAI decreased, and the CDAI remission rate continued to increase from 2012 to 2021. This discordance could be attributed to the difference between DAS28-CRP and CDAI. CDAI criteria are reportedly more stringent than DAS28-CRP criteria, and DAS28-CRP does not correlate with CDAI when patients achieve DAS28 remission [33–36]. Aletaha et al. reported that the sensitivity of CDAI to subtle changes in disease activity (ACR < 20) is higher than DAS28 and Simplified Disease Activity Index (SDAI) [37]. In the KURAMA cohort, approximately 80% of patients achieved DAS28-CRP remission in 2021; thus, DAS28-CRP might not have correlated with CDAI in the current study.

Various treatment outcomes of b/tsDMARDs have been observed in randomized control trials and real-world studies [38–43]. In this study, we analyzed the treatment outcomes in b/tsDAMRD-naïve patients and switched patients and found the following: (1) in naïve patients, TNFi most effectively improved disease activity and functional disability after 3 months, though there were no significant differences in disease activity, functional disability, or drug retention rate between MOAs at 12 months, and (2) in switched patients, IL6Ri yielded significantly lower DAS28-CRP at 12 months and higher retention rate compared to reagents with other MOAs. These findings agree with a recent systematic review where non-TNFi drugs showed better retention rates, and there were no significant differences between bDMARDs in bDMARD-naïve patients [44]. We also observed lower concomitant usage of MTX in IL6Ri users. This trend may reflect the recommendations for RA treatment [45, 46]. The lower concomitant use of MTX and higher concomitant use of GCs in the CTLA4-Ig group may reflect the differences in patient characteristics such as age, comorbidities, and MTX intolerance [47].

Regarding drug persistence, IL6Ri had a better drug retention rate in the KURAMA cohort. Our findings are consistent with previous studies reported by Li et al., who assessed the treatment outcomes of over 8000 patients and observed the superior drug persistence of IL6Ri. Ebina et al. and Jinno et al. also found that IL6Ris had a better retention rate in the ANSWER cohort, a multicenter observational RA cohort where the KURMA cohort participates [23, 48, 49]. Finally, a systematic review by de Castro et al. showed greater persistence of non-TNFi over TNFi [44]. The improvement of disease activity and functional disability in the KURAMA cohort could be attributed to the evolution of RA treatment strategies during the past decade, in which the usage of b/tsDMARDs increased.

The emergence of b/tsDMARDs has altered RA treatment strategies. In this study, we aimed to assess whether the increased treatment options have indeed benefited RA patients in a real-world setting. To the best of our knowledge, this is the first study that revealed annual improvements in disease activity and functional disability using a mixed-effect model. Although it cannot be certain that the yearly trends in CDAI and HAQ in the mixed-effect models represent the increase in treatment options, some studies support that this may be the case. In the study period, RA refractory to multiple b/tsDMARDs was defined as difficult-to-treat RA [50–53]. Ochi et al. reported that JAKi is preferable for difficult-to-treat RA [54]. The emergence of JAKis and their usage optimization might positively affect RA management. Another factor that could account for the annual decline in disease activity and functional disability is the encouragement of exercise or physical therapy. Exercise is reported to improve functional disability in RA patients [55]. We previously reported that sarcopenia was associated with worse RA management [56]. Advances in treatment and care may have contributed to the annual improvements in disease activity and functional disability.

This study has the following limitations: First, individual patient circumstances, such as comorbidity, allergy history, and socioeconomic status, can affect the choice and outcome of RA treatment, but these factors were not considered here because this study analyzed a relatively large dataset of 5070 RA patients who participated in the annual RA survey and 1816 RA patients who initiated b/tsDMARDs. Given the nature of this real-world observational cohort study, the presence of other, possibly unknown, confounding factors may have influenced the results. Second, the annual RA survey does not include all RA patients who are regularly treated in our hospital.

## Conclusions

In summary, we reviewed the real-world transition of therapeutic strategies and their outcomes in the 10-year history of the KURMA cohort. The disease activity and functional disability metrics of patients with RA improved over a decade with increased use of b/tsDMARDs.

### Supplementary Information

**Additional file 1: S. Table1.** Baseline characteristics of patients with RA who enrolled in the annual RA survey.

**Additional file 2: S. Table 2.** Changes in disease activity and functional disability of the KURAMA cohort over the last decade.

**Additional file 3: S. Table 3.** Baseline characteristics of b/tsDMARDs-initiated patients.

**Additional file 4: Fig. S1.** Trend in b/tsDMARDs prescription. Bar charts showing the percentages of prescribed b/tsDMARDs in each year. **Fig. S2.** Treatment outcomes of b/tsDMARDs. A and B. Averaged treatment response comparing each MOA from baseline to 12 months. Left panel: DAS28-CRP, middle panel: CDAI, right panel: HAQ. Upper dotted line in DAS28-CRP: 2.7, lower dotted line in DAS28-CRP: 2.3, upper dotted line in CDAI: 10, lower dotted line in CDAI: 2.8, dotted line in HAQ: 0.5. A. b/tsDMARD-naïve patients. B. Switched patients. **Fig. S3.** Selection of the initial b/tsDMARDs for naïve patients and MOA selection pattern at the time of switching. Sankey diagram showing retention or switching of b/tsDMARDs initiated in KURAMA cohort for naïve patients, and the choice of MOA at the time of switching. The diagram shows the first through the fourth b/tsDMARDs. MOA: mode of action.

## Data Availability

The datasets used during the current study are available from the corresponding author upon reasonable request.

## Abbreviations

RA: Rheumatoid arthritis
MTX: Methotrexate
bDMARD: Biologic disease-modifying antirheumatic drug
tsDMARD: Targeted synthetic antirheumatic drug
JAKi: Janus kinase inhibitor
KURAMA cohort: Kyoto University Rheumatoid Arthritis Management Alliance cohort
GC: Glucocorticoid
DAS: Disease Activity Score
CDAI: Clinical Disease Activity Index
HAQ: Health Assessment Questionnaire Disability Index
ACR: American College of Rheumatology
ANOVA: Analysis of variance
MOA: Mode of action
IL6Ri: IL-6 receptor inhibitors
TNFi: TNF inhibitors
