# Supplementary material for: Management and treatment outcomes of rheumatoid arthritis in the era of biologic and targeted synthetic therapies: evaluation of 10-year data from the KURAMA cohort
Source: Arthritis Res Ther. 2024 Jan 9;26:16. doi: 10.1186/s13075-023-03251-z (PMC10775516; doi:10.1186/s13075-023-03251-z)
Supplement: Supplementary file 4 — Additional file 4: Fig. S1. Trend in b/tsDMARDs prescription. Bar charts showing the percentages of prescribed b/tsDMARDs in each year. Fig. S2. Treatment outcomes of b/tsDMARDs. A and B. Averaged treatment response comparing each MOA from baseline to 12 months. Left panel: DAS28-CRP, middle panel: CDAI, right panel: HAQ. Upper dotted line in DAS28-CRP: 2.7, lower dotted line in DAS28-CRP: 2.3, upper dotted line in CDAI: 10, lower dotted line in CDAI: 2.8, dotted line in HAQ: 0.5. A. b/tsDMARD-naïve patients. B. Switched patients. Fig. S3. Selection of the initial b/tsDMARDs for naïve patients and MOA selection pattern at the time of switching. Sankey diagram showing retention or switching of b/tsDMARDs initiated in KURAMA cohort for naïve patients, and the choice of MOA at the time of switching. The diagram shows the first through the fourth b/tsDMARDs. MOA: mode of action. [file 13075_2023_3251_MOESM4_ESM.pdf]

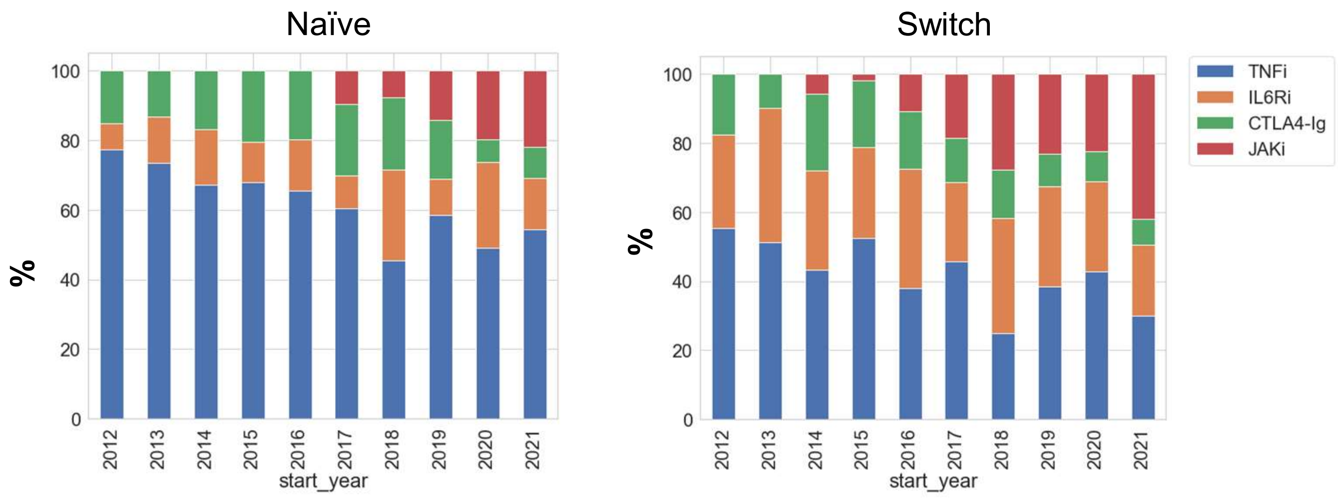

Supplementary Figure. 1. Trend in b/tsDMARDs prescription.
Bar charts showing the percentages of prescribed b/tsDMARDs in each year.

Supplementary Fig. 1

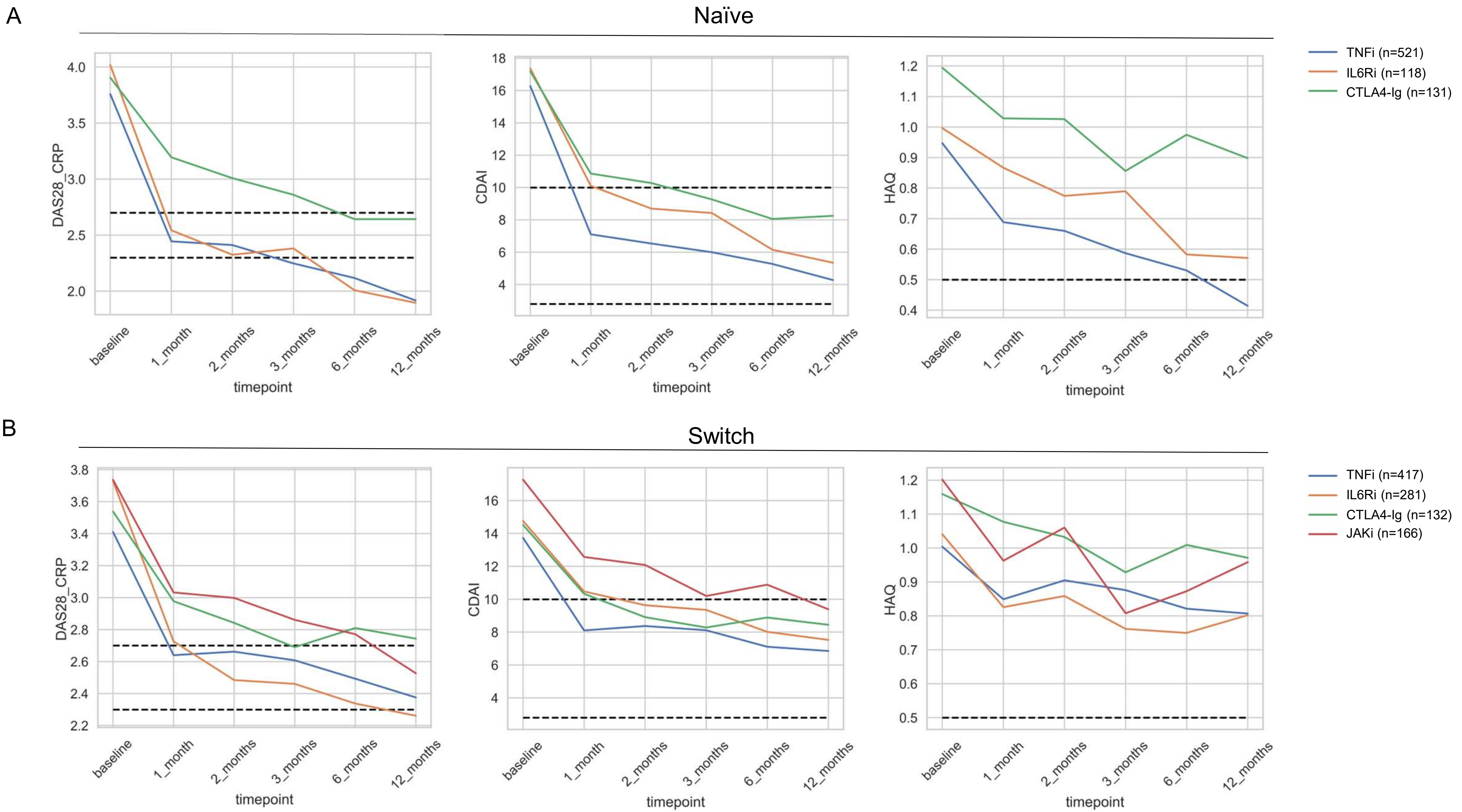

Supplementary Figure 2. Treatment outcomes of b/tsDMARDs.

A and B. Averaged treatment response comparing each MOA from baseline to 12 months. Left panel: DAS28-CRP, middle panel: CDAI, right panel: HAQ. Upper dotted line in DAS28-CRP: 2.7, lower dotted line in DAS28-CRP: 2.3, upper dotted line in CDAI: 10, lower dotted line in CDAI: 2.8, dotted line in HAQ: 0.5. A. b/tsDMARD-naïve patients. B. Switched patients.

Supplementary Fig. 2

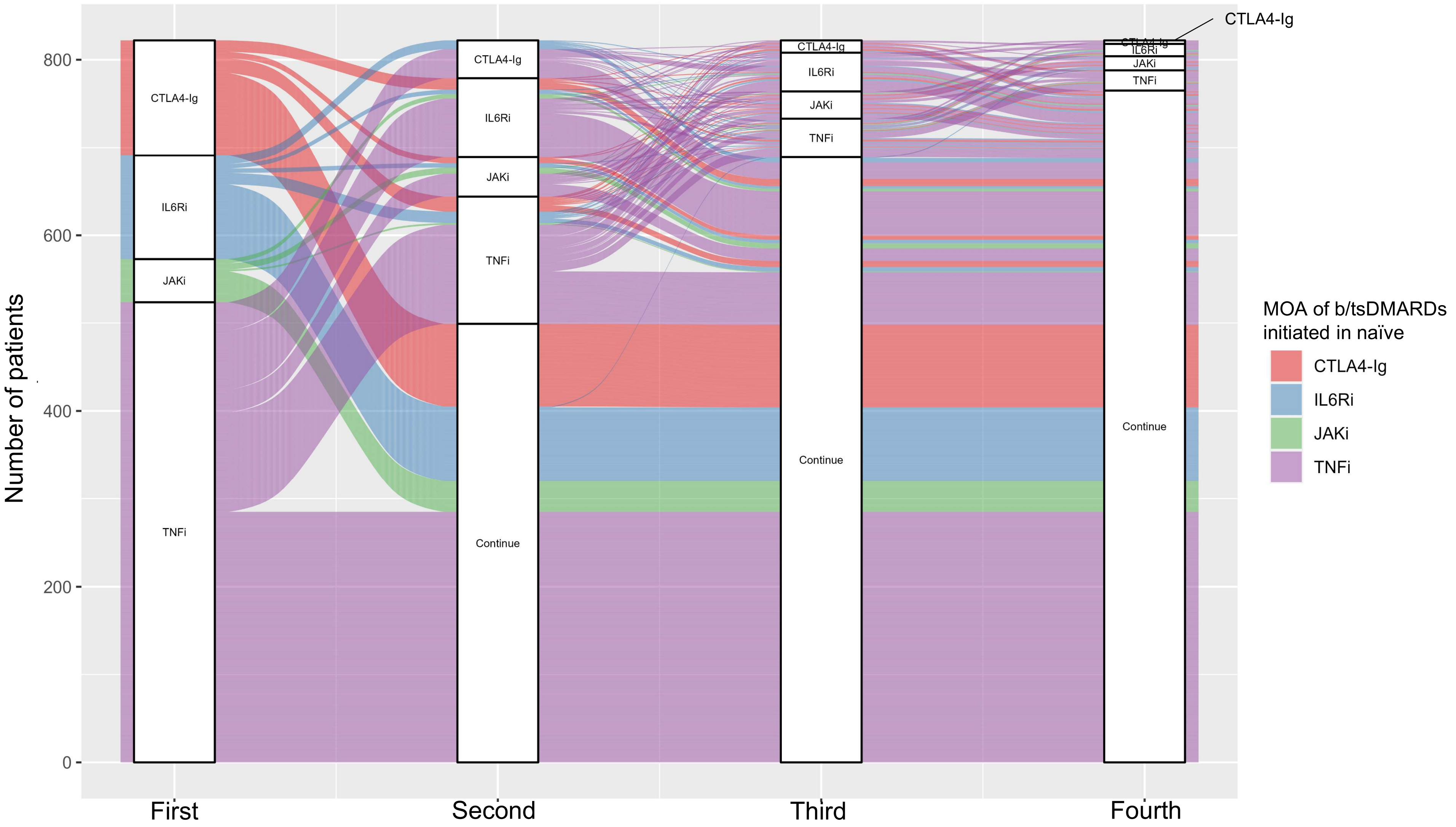

Supplementary Figure 3. Selection of the initial b/tsDMARDs for naïve patients and MOA selection pattern at the time of switching.
Sankey diagram showing retention or switching of b/tsDMARDs initiated in KURAMA cohort for naïve patients, and the choice of MOA at the time of switching. The diagram shows the first through the fourth b/tsDMARDs. MOA: mode of action.

Supplementary Fig. 3
